# Effects of infection control bundle to prevent nosocomial infection in the ICU

**DOI:** 10.1186/cc14163

**Published:** 2015-03-16

**Authors:** A Matsushima, M Kawanami, S Fujimi, N Inadome, N Kubo, T Yoshioka

**Affiliations:** 1 Osaka General Medical Center, Osaka, Japan

## Introduction

Multidrug-resistant organism (MDRO) infections in critically ill patients are often life-threatening. To prevent nosocomial infections of MDRO, we made an infection control bundle in our ICU in 2013. In this study we evaluated the effect of our infection control bundle to prevent nosocomial MDRO transmission and infection.

## Methods

Our infection control bundle consists of preemptive contact precaution to all care, active surveillance culture and isolation of patients with MDRO. This bundle was applied to all patients admitted to our ICU since 2013. The study period to evaluate the effects of the bundle was from April 2012 to March 2014, and we divided it into two periods; first period (before Introduction of the bundle) and second period (after Introduction of the bundle). We compared the incidence of nosocomial transmission and infection of MDRO between the two periods. MDRO was defined as bacteria that were resistant to more than three kinds of antibiotics. Nosocomial transmission was defined when MDRO was detected later than 48 hours after admission. Nosocomial infection was diagnosed according to the National Nosocomial Infection Surveillance Manual.

## Results

Admission to the ICU comprised 363 patients in the first period and 380 patients in the second period. The incidence of transmission was decreased from 48 (13.2%) to 21 (5.5%) in methicillin-resistant *Staphylococcus aureus *(MRSA), from 16 (4.4%) to zero (0%) in multidrugresistant *Acinetobacter baumannii*. The incidence of nosocomial infection by MDRO was also decreased from 23 (6.3%) to 17 (4.5%) in pneumonia, from five (1.4%) to two (0.3%) in urinary tract infection, and from 12 (3.3%) to one (0.3%) in surgical site infection. The incidence of antibiotic use for MDRO infection was decreased from 41 (11.3%) to 24 (6.3%) in anti-MRSA antibiotics, and from 19 (5.2%) to eight (2.1%) in carbapenems.

## Conclusion

Introduction of infection control bundle in the ICU reduced the incidence of nosocomial MDRO transmission and infection, which resulted in the reduction of anti-MRSA antibiotics and carbapenems use in critically ill patients.

